# The diminishing socioeconomic disparity in obesity in a Chinese population with rapid economic development: analysis of serial cross-sectional health survey data 2002–2010

**DOI:** 10.1186/s12889-015-2654-9

**Published:** 2015-12-23

**Authors:** Xiang Qian Lao, WenJun Ma, Roger Yat-Nork Chung, YongHui Zhang, YanJun Xu, XiaoJun Xu, ShaoPing Nie, QiuMao Cai, Liang Xia, XueFen Su, Lei Jin, Tony Tam, Benny Chung-ying Zee

**Affiliations:** School of Public Health and Primary Care, The Chinese University of Hong Kong, Hong kong, China; Shenzhen Research Institute, The Chinese University of Hong Kong, Hong Kong, China; Guangdong Provincial Institute of Public Health, Center for Disease Control and Prevention of Guangdong Province, Guangzhou, China; Center for Disease Control and Prevention of Guangdong Province, Guangzhou, China; Department of Sociology, The Chinese University of Hong Kong, Hong Kong, China

**Keywords:** Social inequality, Obesity, Socio-economic status (SES), Economic development, Chinese

## Abstract

**Background:**

Social changes and economic development are associated with obesity epidemic. This study is to investigate the trends of socio-economic disparity in obesity from 2002 to 2010 in a Chinese population experiencing the world’s fastest economic development.

**Methods:**

Four standardized surveys were conducted in a population of 85 million residents in Guangdong, China between 2002 and 2010. Multistage random cluster sampling was used to recruit representative samples. Information on socio-economic status (SES), proxied by education, occupation and residential area, was collected by face-to-face interviews. The weight, height and waist circumference of the participants were also measured.

**Results:**

Women with low education had an increased BMI of 0.85 kg/m^2^, while women with high education had a decreased BMI of 0.16 kg/m^2^ (*p* = 0.032 for interaction test). Similar trends were observed by using occupation and residential area as the SES indicators. Analysis in men yielded similar patterns. Waist circumference increased from 73.7 to 78.4 cm, and the increasing trends of statistical significance (*p* < 0.01) were observed in both genders and across all SES levels, with the magnitudes of increase in low SES levels being more pronounced. The impact of gradient in food attainment and occupational physical activity across the SES levels may wear off with further economic development, while factors such as health awareness, diet pattern and leisure activity may become increasingly important in driving the disparity.

**Conclusion:**

The impact of gradient in food attainment and occupational physical activity across the SES levels may wear off with further economic development, while factors such as health awareness, diet pattern and leisure activity may become increasingly important in driving the disparity. Our findings suggest that health education should focus on the disadvantaged populations on health awareness for adopting healthier diet pattern and increasing physical activity.

## Background

Obesity is a significant public health challenge worldwide. Rapid economic development and industrialization over the past several decades coincided with an accelerated obesity epidemic, particularly in developing countries [1]. The World Health Organization (WHO) recently estimated that globally over one and a half billion adults are overweight or obese [2]. This translates into a huge obesity-related disease burden in the future.

Socio-economic factors are important determinants of obesity. The relationship between socio-economic status (SES) and obesity has been well summarized by McLaren [3]. Generally, an inverse relationship was observed in high-income countries, while the relationship was positive in low- and mid-income countries. However, the relationship may change over time according to the stage of economic development and industrialization [4]. Caballero [5] pointed out that obesity has been seen more common in lower socioeconomic groups, and this pattern occurred first in developed countries, and more recently in developing countries. In contrast, information concerning changes in the relationship over time is less documented. Data from developed world are inconsistent [6–10], whereas there is little information available from developing countries. On the other hand, developing countries nowadays have a much faster pace of economic development and urbanization than previously experienced in traditionally developed Western populations, leading to accelerated adverse changes in nutrition and lifestyle. Therefore, findings from the developed world may not be as informative. Lack of such information may hinder our understanding of the driving forces behind the accelerated obesity epidemic in the currently developing countries, which is important for the appropriate development of prevention strategies.

China is the world’s largest developing country, and it has had the world’s fastest gross domestic product (GDP) growth rate over the past three decades. Guangdong province is located in southern China with a population of 85 million [11]. Its GDP growth has been the fastest over the past three decades among all the 34 provinces and autonomous regions of China, with an average annual growth rate of 13.6 % [12]. Guangdong therefore provides a unique opportunity to examine the evolvement of social disparity in obesity within the context of rapid economic development. In the present study, we analyzed data on the trajectory of obesity in different SES groups from four standardized cross-sectional health surveys conducted in Guangdong between 2002 and 2010. We investigated the relationships between obesity and SES in each survey and observed changes in the relationship over the survey period. The unique feature of this population (experiencing the world’s fastest economic development) allowed us to compare the difference in the evolvement of social disparity in obesity between the traditionally long-term developed Western populations and populations under more recent and rapid economic development. Findings from the present study will help to understand the mechanisms driving such disparities, and thus may better inform prevention strategies for obesity.

## Methods

### Setting

The Guangdong Health Survey is a series of studies designed to assess the health status of residents in Guangdong. Details of this series of surveys have been described in previous publications [13–17]. Ethical approvals were obtained from the Ethics Committee of the China Center for Disease Control, as well as the Ethics Committee of the Guangdong Provincial Center for Disease Control and Prevention. Written consent was obtained from participants during the interview. Four standardized health surveys were conducted in 2002, 2004, 2007 and 2010 (hereafter referred to as Survey 2002, Survey 2004, Survey 2007 and Survey 2010). Multistage stratified random cluster sampling with probability proportional to size of population was used to recruit representative population samples in these surveys. The sampling details of each survey were described elsewhere [13, 17]. Briefly, cities and counties in the province were categorized into four strata (namely, large cities, small to medium cities, class 1 rural areas, and class 2 rural areas) based on their level of economic development as identified by the central government of China in 1990s [18]. Afterwards, systematic random sampling method was applied to recruit representative population samples in each stratum. Standard sampling protocols were used in all of the four surveys. The sampled populations in each wave of the surveys were different.

For each survey, a central survey site was set up in each selected cluster. At the site, the participants were interviewed face-to-face, and received health examinations on-site. The surveys were conducted by physicians or relevant health professionals who had received training specifically for the surveys, which complied with standard protocols. The survey questionnaires elicited a wide range of information, including demographic characteristics, lifestyle, family, and personal disease histories.

### Socio-economic status measurement

Information on education, occupation and residential area were collected in the present study and were used as indicators of SES. The residence area in Guangdong province was classified into urban and rural areas by the Central Government in the early 1990s based on their economic development levels at the time [18]. The categorization has not changed during the survey period. Participants were grouped into either the urban or rural categories, according to their residential address. Education information was collected during the interview with the following categories: 1) no formal education (zero years); 2) primary school (1 to 5 or 6 years); 3) junior secondary school completed (3 years); 4) senior secondary school or equivalent (3 years); 5) college or above (3 years or above required); and, 6) do not know. Education was then further classified into three categories for analyses: the participants whose education were 1) and 2) were grouped into “up to primary school” (<5 or 6 years of formal education), 3) was grouped into “junior secondary school” (8 or 9 years), and 4) and 5) were grouped into “senior secondary school or above” (more than 10 years). Job information was based on the following question: “Generally your occupation belongs to which of the following categories” with options including 1) workers in agriculture, forestry, stock raising, fishery or water conservancy industries; 2) workers in mining, transportation and manufacturing industries, 3) workers in commercial and services industries; 4) Officials; 5) civil servants or equivalent; 6) professionals; 7) army or related staff; 8) others; 9) students; 10) unemployed; 11) housewives or male house-workers; and 12) retired. During the interview, participants described their job title and the nature of their employment, while interviewers helped to interpret and select the appropriate category. In the data analysis, a participant’s job was being further classified: participants whose jobs were in 1) - 3) were grouped into the “manual” category, 4) - 6) into the “non-manual” category and 7) - 12) into “others.”

### Obesity measurements

Weight and height were measured in the morning before breakfast, with the participants wearing light indoor clothing and no shoes. Waist circumference was measured horizontally around the narrowest circumference between the ribs and the iliac crest. Body mass index (BMI) was calculated as weight in kilograms divided by the square of height in meters. Overweight/overall obesity was defined as BMI ≥25.0 kg/m^2^ based on the WHO suggestions for Chinese [19], while abdominal obesity was defined as waist circumference ≥90 cm in men and ≥80 cm in women based on the guidelines of the International Diabetes Federation [20].

### Statistical analysis

We included only the residents between 18 and 69 years of age in the present analysis, because Survey 2004 and Survey 2007 recruited only residents who were between those ages and we wanted age levels to be consistent across the four surveys. The number of participants with complete information (including age, sex, BMI, waist circumference, education, occupation and residential area) in each of the Surveys in 2002, 2004, 2007, and 2010 included in the present analysis were 12,920, 7609, 6177, and 8541, respectively.

All data analyses were performed using SAS software, version 9.2 (SAS Institute, Cary, NC, U.S.A). Because previous studies have shown the relationships between SES and obesity may be different in men and women [3], we analyzed data separately by sex. Design parameters, including weighting, stratum and cluster, were incorporated into all the analyses because stratified multi-stage cluster sampling with probability proportional to size was used for sampling. Weightings were derived from the 2000 Census data and the associated administrative data [11]. Age-standardized mean or prevalence was calculated by using the age groups 18–34, 35–49, and 50–69 years of the year 2000 Census population. Interaction test was performed to assess the modification effect of SES on obesity overtime (i.e. interaction term “SES*SURVEY YEAR” was included in the models). The odd ratios (ORs) of obesity in different SES groups were calculated for each survey using survey logistic regression adjusting for age. Two-sided *p* values of less than 0.05 were considered to be statistically significant. Standard errors were calculated and presented in the present study.

## Results

### Social demographic characteristics

The mean ages of this population (range 18 to 69) in 2002, 2004, 2007, and 2010 were 44.1, 43.4, 45.1, and 45.2 years, respectively. The distributions of the major socio-economic indicators including education, occupation and residential area in this population stratified by sex are presented in Table 1.

**Table 1:** Socio-demographic characteristics of the residents of 18–69 years of age in Guangdong, 2002–2010

		Survey 2002	Survey 2004	Survey 2007	Survey 2010
		(*n* = 12,920)	(*n* = 7609)	(*n* = 6177)	(*n* = 8541)
Age [mean (se), year]					
All:		44.1 (0.8)	43.4 (0.63)	45.1 (0.73)	45.2 (0.94)
Men:		44.8 (0.9)	43.8 (0.65)	45.2 (0.70)	45.3 (0.89)
Women:		43.6 (0.7)	43.1 (0.70)	45.0 (0.81)	45.0 (1.03)
Education [*n* (%)]					
Women:	Up to primary school	3223 (45.1)	2500 (54.9)	1832 (54.4)	2079 (45.5)
	Junior secondary school	2197 (30.3)	1092 (25.1)	812 (25.2)	1295 (29.8)
	Senior secondary school or above	1814 (24.7)	812 (20.1)	612 (20.3)	1015 (24.7)
Men:	Up to primary school	1477 (27.4)	1105 (34.0)	1091 (36.3)	1372 (32.9)
	Junior secondary school	2086 (35.8)	1203 (36.9)	1068 (36.3)	1516 (36.8)
	Senior secondary school or above	2123 (36.8)	897 (29.0)	762 (27.4)	1264 (30.3)
Occupation [*n* (%)]					
Women:	Manual	3296 (44.7)	2379 (51.9)	1558 (46.2)	1911 (42.1)
	Non-manual	849 (11.4)	434 (10.6)	255 (8.2)	423 (9.8)
	Other	3089 (44.0)	1591 (37.5)	1443 (45.6)	2055 (48.1)
Men:	Manual	2784 (48.9)	1788 (54.2)	1671 (55.8)	2292 (55.0)
	Non-manual	1120 (19.0)	566 (18.3)	363 (12.7)	653 (14.9)
	Other	1782 (32.1)	851 (27.5)	887 (31.5)	1207 (30.1)
Area [*n* (%)]					
Women:	Rural	3730 (50.6)	2663 (58.0)	1910 (55.8)	2512 (55.8)
	Urban	3504 (49.4)	1741 (42.1)	1346 (44.2)	1877 (44.3)
Men:	Rural	3155 (54.5)	2041 (61.8)	1855 (61.0)	2634 (62.6)
	Urban	2531 (45.5)	1164 (38.2)	1066 (39.0)	1518 (37.4)

Percentages are weighted percentages

### Trends in the disparity of BMI and overweight/overall obesity across SES

**Fig. 1:**
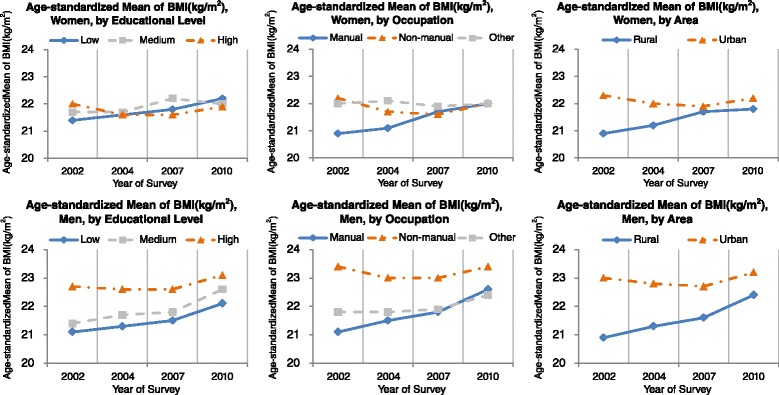
Trends in the age-standardized mean of body mass index (BMI, kg/m^2^) among the residents of 18–69 years of age in Guangdong, 2002–2010 by socioeconomic indicators

The changing trends of age-standardized BMI stratified by SES and sex were presented in Fig. 1. Overall, the age-standardized BMI increased slightly from 21.7 in 2002 to 22.3 kg/m^2^ in 2010 with marginal significance (*p* = 0.062). Among the three groups with different education levels during the survey period of 2002–2010, women with “up to primary school” had the highest average BMI increase of 0.85 kg/m^2^ with a marginal significance (*p* = 0.051). Women with “junior secondary school” had a slight average increase of 0.27 kg/m^2^ without statistical significance (*p* = 0.24). Women with “senior secondary school or above” had a decreased average BMI of 0.16 kg/m^2^, albeit not significant (*p* = 0.62). In each survey, women with “senior secondary school or above” had higher BMI than their counterparts with “up to primary school” in the early stage of the survey period, but the difference decreased and reversed in the later stage of the survey period (*p* = 0.032 for interaction test). Similar results were observed in stratified analysis by occupation and living area: BMI increased among residents with manual occupation or those living in rural area over the survey period, while BMI decreased among residents with non-manual occupation or living in urban area; differences in BMI between manual and non-manual, or rural and urban area became smaller overtime. Analysis in men yielded similar patterns; however, they were less apparent than patterns observed in women.

**Fig. 2:**
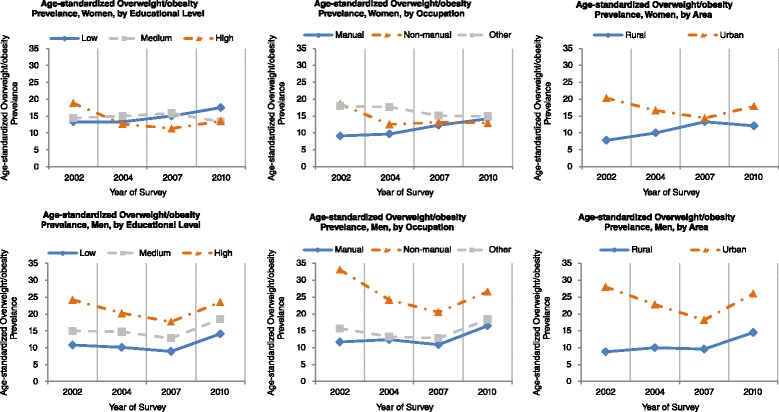
Trends in the age-standardized overweight/obesity among the residents of 18–69 years of age in Guangdong, 2002–2010 by socioeconomic indicators

The changing trends of age-standardized overweight/overall obesity stratified by socioeconomic indicators and sex were presented in Fig. 2. In line with the changing trends of BMI in Fig. 1, the patterns of the changing trends in overweight/overall obesity in each stratum and survey were similar to those in BMI.

### Trends in the disparity of waist circumference and abdominal obesity across SES

**Fig. 3:**
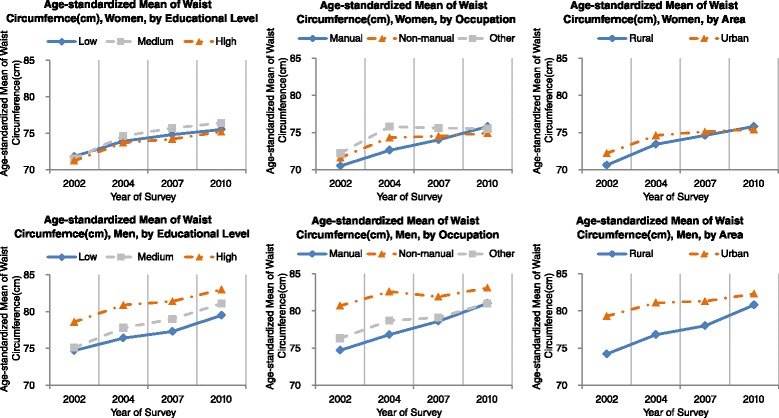
Trends in the age-standardized mean of waist circumference (cm) among the residents of 18–69 years of age in Guangdong, 2002–2010 by socioeconomic indicators

The changing trends of age-standardized waist circumference stratified by socioeconomic indicators and sex were presented in Fig. 3. Overall, the waist circumference increased significantly from 73.7 cm in 2002 to 78.4 cm in 2010. When data were stratified by education level, waist circumference increased significantly in all three education level groups during the survey period in both men and women (*p* ranged from 0.002 to <0.001), and the increased magnitudes were similar among the three education groups (*p* = 0.72 for interaction test). In each survey, waist circumference did not differ among the three education levels in women, but men with “senior secondary school or above” had larger waist circumferences than those with “junior secondary school” and “up to primary school”. When data were stratified by occupation or residential area, apparent increasing trends were observed in each stratum over time in both men and women (all *p* <0.01), but the magnitudes were more pronounced in the manual and the rural strata. In each survey, residents with non-manual occupation and those living in urban area generally had larger waist circumferences in both men and women, but the differences decreased overtime (*p* ranged from 0.0093 to 0.22 for interaction test).

**Fig. 4:**
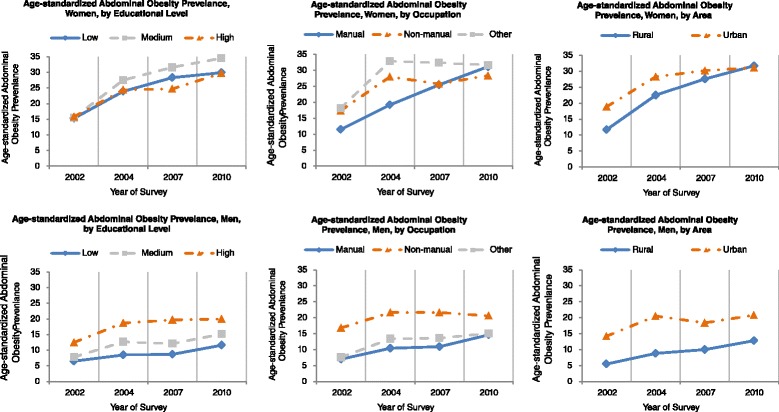
Trends in the age-standardized abdominal obesity among the residents of 18–69 years of age in Guangdong, 2002–2010 by socioeconomic indicators

The changing trends of age-standardized abdominal obesity by all three socioeconomic indicators and sex were presented in Fig. 4. In line with the results of the changing trends in waist circumference in Fig. 3, the patterns of the changing trends in abdominal obesity in each stratum and survey were similar to those changes in waist circumference.

### Trends in the association between obesity and SES

Table 2 shows the ORs between socioeconomic indicators and overall obesity stratified by sex. In women, higher education level was associated with increased risk of overall obesity in Survey 2002, but the association decreased and became non-significant in the later survey stages. For both men and women, residents with non-manual occupation or living in rural area had higher risk of overall obesity, but the associations generally decreased overtime.

**Table 2:** The association between socioeconomic indicators and overall obesity in the residents of 18–69 years of age in Guangdong by sex, 2002–2010

		Survey 2002	Survey 2004	Survey 2007	Survey 2010
Education					
Women	Up to primary school	1.00	1.00	1.00	1.00
	Junior secondary school	1.35 (1.06, 1.69)	1.30 (0.94, 1.81)	1.27 (0.97, 1.66)	0.89 (0.67, 1.19)
	Senior secondary school or above	1.81 (1.27, 2.58)	1.06 (0.82, 1.37)	0.85 (0.59, 1.25)	0.91 (0.64, 1.30)
Men	Up to primary school	1.00	1.00	1.00	1.00
	Junior secondary school	1.59 (1.12, 2.27)	2.16 (1.36, 3.42)	1.63 (1.21, 2.21)	1.53 (1.16, 2.00)
	Senior secondary school or above	3.03 (2.08, 4.40)	1.44 (1.03, 2.03)	2.70 (1.77, 4.12)	2.25 (1.52, 3.33)
Occupation					
Women	Manual	1.00	1.00	1.00	1.00
	Non-manual	2.24 (1.72, 2.91)	1.37 (0.78, 2.41)	1.17 (0.52, 2.61)	0.99 (0.58, 1.70)
	Other	2.41 (1.78, 3.28)	2.23 (1.65, 3.02)	1.61 (1.23, 2.11)	1.37 (1.05, 1.78)
Men	Manual	1.00	1.00	1.00	1.00
	Non-manual	4.19 (3.24, 5.42)	2.39 (1.55, 3.67)	2.60 (1.57, 4.30)	2.14 (1.48, 3.10)
	Other	1.87 (1.16, 3.03)	1.47 (1.07, 2.01)	1.71 (1.19, 2.45)	1.51 (1.10, 2.07)
Area					
Women	Rural	1.00	1.00	1.00	1.00
	Urban	3.30 (2.27, 4.81)	2.13 (1.47, 3.09)	1.45 (0.96, 2.19)	1.97 (1.28, 3.04)
Men	Rural	1.00	1.00	1.00	1.00
	Urban	4.40 (2.87, 6.74)	2.84 (1.87, 4.30)	2.48 (1.75, 3.52)	2.35 (1.52, 3.63)

*The ORs were calculated by using survey logistic regression adjusting for age

Table 3 shows the ORs between socioeconomic indicators and abdominal obesity stratified by sex. Except for women with “junior secondary school” who had a higher risk of abdominal obesity than women with “up to primary school” in Surveys 2004 and 2010, no significant associations between education level and abdominal obesity were observed in women. In men, however, higher education was consistently associated with higher risk of abdominal obesity over the survey period. When occupation and residential area were used as the SES indicators, the associations decreased overtime in both men and women.

**Table 3:** The association between socioeconomic indicators and abdominal obesity in the residents of 18–69 years of age in Guangdong by sex, 2002–2010

		Survey 2002	Survey 2004	Survey 2007	Survey 2010
Education					
Women	Up to primary school	1.00	1.00	1.00	1.00
	Junior secondary school	1.09 (0.96, 1.23)	1.27 (1.00, 1.62)	1.15 (0.91, 1.45)	1.21 (1.02, 1.44)
	Senior secondary school or above	1.06 (0.77, 1.47)	1.04 (0.79, 1.37)	0.78 (0.53, 1.14)	0.95 (0.71, 1.27)
Men	Up to primary school	1.00	1.00	1.00	1.00
	Junior secondary school	1.29 (0.90, 1.84)	1.63 (1.14, 2.34)	1.50 (1.17, 1.93)	1.50 (1.08, 2.09)
	Senior secondary school or above	2.19 (1.49, 3.21)	2.60 (1.54, 4.39)	2.78 (2.15, 3.59)	2.16 (1.27, 3.69)
Occupation					
Women	Manual	1.00	1.00	1.00	1.00
	Non-manual	1.58 (1.28, 1.94)	1.73 (1.13, 2.67)	0.99 (0.48, 2.05)	0.88 (0.60, 1.28)
	Other	1.95 (1.46, 2.60)	2.35 (1.76, 3.15)	1.72 (1.24, 2.38)	1.30 (1.05, 1.60)
Men	Manual	1.00	1.00	1.00	1.00
	Non-manual	3.02 (2.46, 3.70)	2.73 (1.91, 3.90)	2.60 (1.86, 3.64)	1.75 (1.16, 2.64)
	Other	1.78 (1.18, 2.67)	1.82 (1.16, 2.85)	1.82 (1.29, 2.56)	1.42 (0.92, 2.20)
Area					
Women	Rural	1.00	1.00	1.00	1.00
	Urban	2.02 (1.28, 3.17)	1.58 (0.94, 2.65)	1.37 (0.85, 2.21)	1.19 (0.78, 1.82)
Men	Rural	1.00	1.00	1.00	1.00
	Urban	3.21 (2.22, 4.65)	2.94 (1.83, 4.71)	2.35 (1.49, 3.70)	2.06 (1.19, 3.56)

*The ORs were calculated by using survey logistic regression adjusting for age

## Discussion

### Summary of findings

Overall, high SES residents had slightly decreasing trends in BMI and overweight/overall obesity while low SES residents had apparent increasing trends over the observed period from 2002 to 2010. With regards to waist circumference and abdominal obesity, increasing trends were observed in residents of all SES groups, and more dramatically in low SES residents. The disparity in BMI, waist circumference, overweight/overall obesity and abdominal obesity across different SES groups diminished over the observed period. Although the trends in each SES group varied, the age-adjusted mean of BMI/waist circumference and the prevalence of overall/abdominal obesity tended to converge. The associations between SES and overall/abdominal obesity indicated by ORs generally decreased over time.

### Comparison with similar studies

There is relatively less information in the literature on the associations between SES and waist circumference/abdominal obesity, as well as the changing trends in the associations. Our results show the associations and their corresponding trends are similar to those for overall obesity using occupations and residential areas as SES indicators, but the patterns occurred a bit later. The lagged pattern is in line with previous finding on the differential trends in overall and abdominal obesity. Before the 2000s, secular increasing trends in obesity were generally observed worldwide [21]. A temporal association between socioeconomic development and obesity has also been suggested in a population-based study in Hong Kong, the first rapidly developed Chinese population [22]. After the 2000s, increasingly more studies showed that overall obesity epidemic started to plateau in some countries and regions with high socio-economic status [21, 23–25]. However, the trends in abdominal obesity continued to rise despite the leveling off or decreasing trends in overall obesity [26, 27]. The mechanism behind the differential trends in overall and abdominal obesity is unclear. Our results also show that waist circumference/abdominal obesity increased significantly in all SES groups; however, the increases of waist circumference/abdominal obesity in high SES residents were slower than those in low SES residents.

The change of socio-economic disparity in overall obesity varied. Some studies showed no change or a widening social disparity in obesity over time [6–8], while some studies found a diminishing disparity [9, 10, 28]. Nonetheless, most studies showed that the trends in prevalence of obesity across SES groups were similar despite the increasing or decreasing disparity (i.e., the prevalence of obesity increased or plateaued simultaneously in all SES groups despite different magnitudes) [6, 8–10, 28]. Opposite changing trends in BMI/overall obesity between high and low SES groups in our population was observed, within a short period (8 years in the present study). The rapidly diminishing disparity of obesity between high and low SES groups in our population may reflect the impact of much faster economic development.

### Explanations of the findings

The diminishing disparity across SES groups over the survey period and various trends in each SES groups in the present study were consistent with the Epidemiologic Transition Theory proposed by Omran [29], which suggests that in the early stages of social and economic development, a high prevalence of chronic disease is most apparent among the most educated and wealthy, and such trend would slow down or even reverse in later stages as people realize the health hazards of poor diet and lifestyle choices. It has been observed that the chronic disease burden then shifts to poor people in later stage of epidemiologic transition [30]. The susceptibility of high SES residents in the early stages may reflect their capacity to afford and demand surplus food. The high SES residents are also more likely to engage in non-manual occupation, resulting in the expenditure of less energy [31]. But with further economic development, food shortage is no longer a common problem in a society [32]. A higher degree of urbanization and industrialization also renders occupation less laborious even for low SES residents [1, 32]. At this transition stage, factors including health awareness and attitude, the choice of healthy food and leisure-time physical activity may gradually become more important drivers in the differences in obesity among different SES groups. Hence, lower SES residents are more susceptible to the risk of obesity, given their lower levels of health awareness as well as the lower capacity to access healthy food [33]. On the other hand, high SES residents are more likely to become health-conscious earlier, and tend to be in a better financial position to invest in healthy diet and exercise to prevent themselves from becoming obese [31], which may result in a deceleration of the increasing trends or even reversal in trends in obesity for these groups.

### Limitations

There are caveats in this study. First, this study used a relatively short survey period, which prohibited us from drawing a more comprehensive picture of the associative trends between social economic disparity and obesity over time. We observed that the means and prevalence in different SES groups tend to converge. As the directions of socio-economic disparity in obesity are opposite between developing and developed countries [3], we speculate that the trends will continue to converge and finally result in a reversed disparity (i.e., lower SES groups having higher prevalence of obesity than higher SES groups) if there is no interventions to prevent the trends. Second, we only included three factors as proxy indicators of SES (i.e., education, occupation and residence area). While education, occupation and income are traditional indicators used to proxy a person’s SES, income was not used in the present study since it is a rather personal piece of information and thus, a relatively large number of missing data, and the format of the income information was not consistent across the various surveys used. On the other hand, we used residence area as a proxy of SES in this study. It is a unique indicator of SES in China, especially in early time due to the sharp divide between urban and rural areas in terms of income, health care, quality of education, access to public goods such as housing, sanitation, and other dimensions of welfare [34, 35]. The categorization of residence area used in the present study was defined in the 1990s based on the levels of economic development at that time, and has not been changed since then (reference). On the other hand, with rapid economic development, some areas defined in the 1990s as rural are no longer rural today, possibly making residence area an inappropriate SES indicator today in China [36]. Nevertheless, this provided us a unique opportunity to observe the change in social gradient under rapid economic development and its impact on obesity disparity. The differences in food attainment and occupational physical activity between urban and rural areas decreased dramatically over the survey period with rapid urbanization. This may explain the diminishing gaps in obesity between urban and rural residents in our study. Similarly, we deduce the differences also diminish over time between the different SES groups using education and occupation as SES indicators.

## Conclusion

Our findings may have important public health implications as economic growth is a highly desirable and necessary goal for many developing countries. With further economic development, obesity will become a more challenging health threat in the developing world in the coming decades. Overall obesity may continue to increase especially in low SES groups, while it may remain in a high level (might increase in a slower pace, level off or decrease slightly) in high SES groups. The abdominal obesity may catch up quickly in all SES groups, but the increase may be more pronounced in low SES groups. Since abdominal obesity is regarded as more harmful than overall obesity, prevention strategies should put more emphasis on abdominal obesity on the disadvantaged populations [37–39]. On the other hand, with further economic development, the difference in food attainment and occupational physical activity across SES levels may decrease, causing the diminishing social disparity in obesity. In the future, the gradient in health awareness, diet pattern and leisure-time physical activity across SES levels may become the major forces in driving the disparity in obesity, which are similar to those we currently observe in the developed world. Therefore, health education should target the disadvantaged populations on their health awareness for adopting healthier diet pattern and increasing leisure-time physical activity.
